# National, Regional and Global Certification Bodies for Polio Eradication: A Framework for Verifying Measles Elimination

**DOI:** 10.1093/infdis/jiw578

**Published:** 2017-07-01

**Authors:** S. Deblina Datta, Rudolf H. Tangermann, Susan Reef, W. William Schluter, Anthony Adams

**Affiliations:** 1 Global Immunization Division, Centers for Disease Control and Prevention, Atlanta, Georgia;; 2 World Health Organization , Geneva, Switzerland;; 3 Western Pacific Regional Office/World Health Organization, Manila, Philippines; and; 4 Chair, Global Certification Commission for Polio Eradication and Chair, Western Pacific Certification Commission for Polio Eradication

**Keywords:** polio eradication, polio endgame, polio legacy, Global Polio Eradication Initiative, polio, Regional Certification Commission, Global Certification Commission, measles, rubella, Measles and Rubella Initiative, measles elimination.

## Abstract

The Global Certification Commission (GCC), Regional Certification Commissions (RCCs), and National Certification Committees (NCCs) provide a framework of independent bodies to assist the Global Polio Eradication Initiative (GPEI) in certifying and maintaining polio eradication in a standardized, ongoing, and credible manner. Their members meet regularly to comprehensively review population immunity, surveillance, laboratory, and other data to assess polio status in the country (NCC), World Health Organization (WHO) region (RCC), or globally (GCC). These highly visible bodies provide a framework to be replicated to independently verify measles and rubella elimination in the regions and globally.

As of 2016, there has been considerable progress on the 1988 World Health Assembly Declaration on global polio eradication; only 3 countries (Nigeria, Pakistan, and Afghanistan) remain endemic for wild polio virus (WPV). In 2015, there were a total of 74 cases of WPV (down from an estimated 350000 in 1988). The last indigenous WPV type 2 (WPV2) case was identified in 1999 and WPV2 was declared eradicated in September 2015; the last WPV type 3 (WPV3) case was identified in November 2012 [1]. Despite the recent identification of 4 WPV type 1 (WPV1) cases in northeastern Nigeria as of October 2016, there are the fewest number of WPV types in circulation, and the fewest number of polio cases in the fewest number of polio-affected countries. With eradication as an achievable goal in the short term, the process has begun for transitioning polio-related assets, primarily housed within the Global Polio Eradication Initiative (GPEI), into assets for other global health-related goals, such as (1) measles and rubella elimination, and (2) increasing routine immunization coverage.

A critical nontangible asset within GPEI is the formal and standardized process by which polio eradication is certified. This process is based on smallpox eradication certification whereby independent experts were convened to review predetermined sets of criteria for each country involved in smallpox eradication. The smallpox expert commission went through a rigorous review and decision-making process, resulting in a certification report to the World Health Organization (WHO) Director-General and World Health Assembly [2].

The first WHO Region to certify as polio-free was the Region of the Americas (AMR) in 1994. The Regional Office for the Americas convened a Regional Certification Commission in 1990 to oversee the certification process, which used data on surveillance for laboratory-confirmed acute flaccid paralysis (AFP) to determine that polio had been eradicated in the Americas in 1994, after the last case of wild polio occurred in Peru in 1991. The certification process for polio eradication in all WHO regions is similar, with 3 other regions already certified as wild polio–free: Western Pacific Region (WPR) in 2000, European Region (EUR) in 2002, and Southeast Asian Region (SEAR) in 2014. Regional Certification Commissions (RCCs) have been established in the African Region (AFR) and Eastern Mediterranean Region (EMR) and are fully functional in preparation for polio-free certification in the future [3]. The importance of the certification framework is in making independent, timely, evidence-based, standardized, and credible conclusions on the status of polio eradication for the GPEI. This paper will detail the structure, composition, and history of the polio certification process, and describe how it has already begun to be used for the validation of future global health goals as well as leaving open the possibility of additional future applications.

## ROLES AND STRUCTURE OF CURRENT POLIO CERTIFICATION FRAMEWORK

The certification framework is similar in all WHO Regions and is composed of 3 functional bodies at different levels of responsibility: National Certification Committees (NCCs), Regional Certification Commissions (RCCs), and a Global Certification Commission (GCC). The GPEI has ensured that members of RCCs and the GCC are independent of GPEI and commissioned as independent experts by WHO. In addition, there are an agreed-upon set of terms and roles: agreement on basic definitions (including surveillance and other key terms), establishment of principles, and establishment of criteria for eradication.

NCC members are appointed by national polio programs as independent experts to review program performance through a detailed annual report to the RCC. Membership is composed of clinicians (typically, neurologists or infectious disease specialists), virologists, and public health experts in polio from the respective country. In rare instances where country capacity may be limited, members of the NCCs may perform double duty as Polio Expert Review Committee members tasked with reviewing AFP cases for which a diagnosis of polio cannot be ruled out virologically because of inadequate collection of stool specimens.

RCCs consist of independently appointed commissioners with global reputations as polio experts who are tasked with reviewing documentation provided by NCCs and certifying their regions as polio free. It is important to note that “polio-free” status of the WHO region is conferred by RCCs, not by NCCs. In other words, NCCs can report on the status of a country’s polio program but cannot certify a country to be polio-free, highlighting their function as committees as opposed to commissions at regional and global levels. Only regions can be certified “polio free,” not countries. Once all 6 regions are certified, the GCC (which is composed of the 6 chairs of the RCCs) is able to certify the world as free of wild poliovirus and declare global eradication as completed. While NCCs and RCCs meet annually, the GCC meets on an ad hoc basis for global-level decision making, as in its last meeting in 2015 when it declared global eradication of WPV2. This declaration paved the way for withdrawal of trivalent oral polio vaccine (tOPV) globally [4].

Terms of Reference among NCCs are very similar and the main objective is to assemble, review, and submit to the RCC the final national documentation of polio-free status of their country. After achieving polio-free status, the NCCs submit annual updates on maintaining polio-free status. The report touches upon every aspect of the polio program, including surveillance, routine immunization performance, laboratory performance, biocontainment, supplementary immunization activities (SIAs), and Risk Assessments, and even performance of the NCC itself. Table 1 details these report elements. The NCC also makes recommendations about risk mitigation and corrective actions for the polio programs. National polio programs receive this feedback from NCCs and perform recommended actions where necessary.

**Table 1.** Types of Information Reported to Regional Certification Commissions by National Certification Committees

Quality of AFP Surveillance	Laboratory Activities	Quality of Routine Immunization Coverage
*Brief description of adequacy of national AFP surveillance, changes, new projects, program performance*	*Latest accreditation results and corrective measures taken, if any*	*Administrative coverage with DTP3 from national and subnational levels*
*Completeness and timeliness in identifying new AFP cases*	*Summary of all specimens received for poliovirus study*	*Relevant coverage survey data*
*Number and distribution of AFP surveillance reporting sites*	*Completeness of all specimen processing and results reporting*	*Data management*
*Reporting completeness*	*Details on intratypic differentiation results*	*Coverage improvement plans*
*Non-polio AFP rates from national and subnational levels*	*Details on all sequencing results*	Supplementary immunization activities
*Areas with subnational surveillance and actions taken*	*Problems in lab performance and corrective actions taken, if any*	
*Identification and monitoring of silent areas*		Risk assessments
*Cluster and risk analysis of AFP cases*	VDPV surveillance	
*Stool sample collection adequacy and timeliness*	*Vaccine-associated paralytic polio cases*	National action plans for detection of and response to WPV and circulating VDPV
*Work of National Polio Experts Group including documentation of all AFP cases reviewed and final disposition; documentation of any polio compatible cases*	*Detailed information on any VDPV or suspected VDPV associated with AFP cases*	…
*Results of any AFP surveillance reviews conducted*	*Investigation details*	Status of laboratory containment of WPV infectious materials and potentially infectious materials
	*Immunization response and results*	
Description and results of any supplementary polio surveillance (eg, enterovirus surveillance, environmental surveillance, stool surveys)	…	Injectable poliovirus Introduction and implementation of tOPV-bOPV Switch Plan

Abbreviations: AFP, acute flaccid paralysis; bOPV, bivalent oral polio vaccine; DTP3, diphtheria-tetanus-pertussis; tOPV, trivalent oral polio vaccine; VDPV, vaccine-derived polio virus, WPV, wild polio virus.

The 6 RCCs have primarily served under similar Terms of Reference. Of particular note is the reconstitution of the AMR RCC in 2014 after a period of dormancy since the 1994 certification of the region as polio free. The AMR RCC in its current form has been shaped to address current needs within GPEI, and is officially titled the “Regional Commission on the Certification of the Polio Endgame,” which explicitly includes the additional areas of the tOPV to bivalent oral polio vaccine (bOPV) global switch and biocontainment. The other 5 RCCs function quite similarly to each other. While these 5 do not have an official role in the tOPV-to-bOPV switch, each has played an important facilitation and advocacy role in the successful, globally coordinated switch [5–8]. Once regional certification is achieved, RCCs play critical roles in maintaining the region free of polio until global certification by:

1. providing a strong advocacy voice for eradication activities to countries in the region;
2. identifying ongoing or upcoming threats for polio outbreaks in the regions; making risk mitigation and corrective action recommendations; and
3. providing oversight over ongoing GPEI priorities (eg, containment, global switch from tOPV to bOPV, introduction of inactivated poliovirus vaccine, prevention and response to any emergence of vaccine-derived polioviruses [VDPVs], interruption of WPV transmission, and the function of laboratory networks).

## TRANSITIONING POLIO CERTIFICATION FOR FUTURE GLOBAL IMMUNIZATION NEEDS

The main strength of the polio certification framework lies in its credibility to accurately assess when WPV has been eradicated. This model of certification of disease eradication can be used in transitioning assets out of polio-related functions in 2 manners: (1) using the certification framework as a model for certifying the eradication and elimination of other diseases, and (2) using specific resources; in this case, trained human resources serving on NCCs and RCCs as members of other expert committees for immunization or child health–related activities, especially in human resource–constrained countries (assuming the members have required relevant additional expertise; eg, measles). The first manner of transitioning assets has already begun by the Measles Rubella Initiative in verifying measles and rubella elimination.

### Utilizing the Polio Certification Model in Constructing a Measles/Rubella Verification Framework

In 2013, a seminal guidance paper was published outlining the development of a framework for verifying measles/rubella elimination, which was endorsed by the Strategic Advisory Group of Experts on Immunization (SAGE) [9]. In it, WHO outlined the following framework components for regions in which elimination targets have been determined, borrowing heavily from the polio eradication certification framework: establishing multidisciplinary National Verification Committees (NVCs) at the country level to “gather, analyze, and validate the national data, and submit the necessary documentation to the Regional Verification Commission (RVC).” RVCs are to be established as independent commissions staffed with recognized experts, and are tasked with the annual progress review toward measles and rubella elimination for each individual country or area in the region. RVCs may declare regional measles and rubella elimination when all countries have documented ≥36 months of interrupted endemic measles and rubella transmission.

Most importantly, the document establishes the 3 criteria for verifying measles and rubella elimination, which include the documentation of the interruption of endemic measles and rubella virus transmission for a period of at least 36 months from the last known endemic case, and the presence of a high-quality surveillance system and genotyping evidence that supports interruption of endemic transmission. The document also establishes the lines of evidence that allow for a comprehensive evidence-based assessment of program performance, which include the 3 criteria mentioned above in addition to other supportive information, allowing for a comprehensive assessment of past program performance and future capacity to sustain elimination. Supportive information includes:

1. a detailed description of the epidemiology of measles and rubella since the introduction of measles and rubella vaccine in the national immunization program;
2. population immunity, presented as a birth cohort analysis with the addition of evidence related to any marginalized and migrant groups;
3. quality of epidemiological and laboratory surveillance systems for measles and rubella;
4. sustainability of the national immunization program, including resources for mass campaigns, where appropriate, to sustain elimination; and
5. genotyping evidence that measles and rubella virus transmission has been interrupted.

Table 2 summarizes the analogies between certification of polio eradication and verification of measles and rubella elimination, and succinctly demonstrates how the measles and rubella verification framework was able to build upon the strength of the polio certification framework.

**Table 2.** Analogies Between Certification and Verification Frameworks for Polio and Measles/Rubella

	Polio	Measles/Rubella
Certification/verification conducted on a regional basis	Yes	Yes
Standardized case definitions	AFP case with stool specimen testing for polio	Acute febrile rash case with serum testing for measles/rubella
Standardized case classification	Yes	Yes
Standardized laboratory testing and reporting protocols	Yes	Yes
Essential criteria for certification/verification	Absence of WPV transmission for >36 months in the presence of certification standard surveillance	Absence of endemic transmission of measles/ rubella for a period of >36 months; high-quality surveillance; genotyping evidence supporting interruption of endemic transmission
National-level certification/ verification bodies	National certification committees	National verification committees
Regional-level certification/ verification bodies	Regional certification commissions (6/6 regions)	Regional verification commissions (4/6 regions)
Global-level certification/ verification bodies	Global certification commission	Global verification commission
Laboratory containment	Yes	No

Abbreviations: AFP, acute flaccid paralysis; WPV, wild polio virus.

As of this writing, 4 of 6 WHO Regions have established RVCs, including: EURO (first meeting, 2012), WPR (2012), SEAR (2016), and AMR (2010). The regions have appointed national- and regional-level committee/commission members, and are convening regular meetings. The RVCs have made excellent progress in engaging with NVCs and orienting them on preparing national documentation, reviewing elimination criteria and guidelines, and establishing terms of reference. Several RVCs have conducted training workshops for NVC members [10, 11].

### Utilizing Polio Certification Assets for Other Child Health Programs

The usefulness of transitioning polio assets by using the experienced corps of NCC and RCC members has yet to be determined. Several important expert immunization committees exist at the national level, including NVCs, National Immunization Technical Advisory Groups (NITAGs), and Interagency Coordination Committees (ICCs). In resource-constrained countries, the Supporting Independent Immunization and Vaccine Advisory Committees (SIVAC) Initiative, a group that supports countries in developing and strengthening NITAGs, has identified a lack of sufficiently experienced and adequately trained experts to serve on NITAGs [12]. NITAGs are charged with the task of providing recommendations on immunization policies and programs, including immunization schedules, improvement of routine immunization coverage, and introduction of new vaccines. As such, NITAGs are most capable when staffed by in-country experts who are familiar with the local context [13]. A current study is investigating whether NCC and/or RCC members can be transitioned to NITAGs, and whether additional resources will be needed to support these experts in 8 developing countries.

While it is encouraging that the transitioning of polio certification assets has begun (measles and rubella verification), it is clear there are other areas that need to be investigated to fully utilize the well-established experience of the polio certification process in countries, nationally, regions, and globally.

## Notes

***Acknowledgments.*** We thank Dr Mark Papania, MD, Global Immunization Division, Centers for Disease Control and Prevention (CDC); and Dr Walter Dowdle, MD, of the Task Force on Global Health for their careful and helpful review of this paper.

***Disclaimer.*** The findings, interpretations and conclusions expressed in this paper are those of the authors and do not necessarily reflect the policies or views of the World Health Organization (WHO) or CDC.

***Supplement sponsorship.*** This work is part of a supplement coordinated by the Task Force for Global Health with funding provided by The Bill and Melinda Gates Foundation and the Centers for Disease Control and Prevention.

***Potential conflicts of interest.*** All authors: No reported conflicts of interest. All authors have submitted the ICMJE Form for Disclosure of Potential Conflicts of Interest. Conflicts that the editors consider relevant to the content of the manuscript have been disclosed.
